# A systematic review on current status of health technology reassessment: insights for South Korea

**DOI:** 10.1186/s12961-016-0152-x

**Published:** 2016-11-11

**Authors:** Hyun-Ju Seo, Ji Jeong Park, Seon Heui Lee

**Affiliations:** 1 Department of Nursing, College of Medicine, Chosun University, Gwangju, Korea; 2 National Evidence-based Healthcare Collaborating Agency, Seoul, Korea; 3 Department of Nursing Science, College of Nursing, Gachon University, 191 Hambakmoero, Yeonsu-gu, 406-799 Incheon, Korea

**Keywords:** Health technology reassessment, Disinvestment, Systematic review

## Abstract

**Background:**

To systematically investigate the current status and methodology of health technology reassessment (HTR) in various countries to draw insights for the healthcare system in South Korea.

**Methods:**

A systematic literature search was conducted on the articles published between January 2000 and February 2015 on Medline, EMBASE, the Cochrane Library, CINAHL, and PubMed. The titles and abstracts of retrieved records were screened and selected by two independent reviewers. Data related to HTR were extracted using a pre-standardised form. The review was conducted using narrative synthesis to understand and summarise the HTR process and policies.

**Results:**

Forty five studies, conducted in seven countries, including the United Kingdom, Australia, Canada, Spain, Sweden, Denmark, and the United States of America, fulfilled the inclusion criteria. Informed by the literature review, and complemented by informant interviews, we focused on HTR activities in four jurisdictions: the United Kingdom, Canada, Australia, and Spain. There were similarities in the HTR processes, namely the use of existing health technology assessment agencies, reassessment candidate technology identification and priority setting, stakeholder involvement, support for reimbursement coverage, and implementation strategies. Considering the findings of the systematic review in the context of the domestic healthcare environment in Korea, an appropriate HTR model was developed. This model included four stages, those of identification, prioritisation, reassessment and decision.

**Conclusions:**

Disinvestment and reinvestment through the HTR was used to increase the efficiency and quality of care to help patients receive optimal treatment. Based on the lessons learnt from other countries’ experiences, Korea should make efforts to establish an HTR process that optimises the National Healthcare Insurance system through revision of the existing Medical Service Act.

## Background

In South Korea, health expenditures have increased rapidly since the introduction of the national health insurance in the second half of the 1980s. Between 2000 and 2009, the rate of increase in health spending in South Korea was more than twice the average across OECD countries, reaching and average of 9.8% per year in real terms, compared to that of 4.8% in OECD countries [1]. Although there has been an improvement in the assessment of new health technologies in Korea since the introduction of a formal assessment procedure in 2007, an efficient mechanism to promote the reassessment and management of obsolete health technologies is yet to be established. In particular, there have been rising concerns about new technologies introduced to the market after being evaluated in new health technology assessment (HTA) programs with regards to patients’ safety and the efficiency of the National Health Insurance system. Leggett et al. [2] reported that there were some uncertainties regarding the safety and effectiveness of new health technologies and the obsolescence of existing technologies because of their lifecycle. Therefore, reassessment of their safety and effectiveness has been required since the introduction of the new technology under health insurance coverage. In the 2012 Health Policy Forum of Health Technology Assessment International [3], health technology reassessment (HTR) was proposed as an integral part of HTA agencies in order to enhance the optimal use of health technologies and to ensure value for money.

In December 2008, the National Evidence-based healthcare Collaborating Agency (NECA) was established to take charge of HTA and economic evaluation research in South Korea. Subsequently, in 2010, the Center for New Health Technology Assessment was integrated into the NECA [4]. Recently, several countries, such as the United Kingdom, Australia, Canada, and Spain, have demonstrated growing interest in HTR development and implementation. However, there was no systematic literature review of HTR to determine its current status and provide insight for other countries planning to introduce a similar system. Therefore, the aim of this study was to systematically review the HTR-related literature to examine (1) the organisation/governance of HTR; (2) the process used for identifying candidate technology for reassessment and candidate technology prioritisation; (3) the stakeholder engagement, decision-making processes, and implementation strategies; and (4) the general HTR landscape to obtain insights into developing HTR in South Korea.

## Methods

### Literature search and inclusion criteria

We searched the literature published from January 2000 to February 2015 using electronic databases including Medline, EMBASE, the Cochrane Library, CINAHL, and PubMed. In order to develop a comprehensive search strategy, we performed the pilot search using MeSH terms or keywords mentioned in key references related to HTR. We conducted the search using a comprehensive combination of keywords, such as “disinvestment”, “obsolescence”, “ineffective”, “reassessment”, “reallocation”, “program budgeting”, “abandoned”, “optimal practice or use”, “health investment or reinvestment”, and “value for money” (Table 1). Thirty-two of the 53 Health Technology Assessment International (HTAi) and International Network of Agencies for Health Technology Assessment (INAHTA) members had accessible English websites, which were searched to identify related unpublished reports. Additionally, other relevant studies were searched by manually screening the reference lists of the studies included in the search.

**Table 1:** Definitions

Health technology disinvestment	“*The complete or partial removal of a health technology based on evidence that it is clinically ineffective and/or financially inefficient*” [2]. “*The process of withdrawing (partially or completely) health resources from those existing healthcare practices, procedures, technologies, and pharmaceuticals that are deemed to deliver no or low health gain and are thus not efficient health resource allocations*” [36]
Health technology reassessment	“*A structured, evidence-based assessment of the clinical, social, ethical & economic effects of a technology currently used in the health care system, to inform optimal use of that technology in comparison to its alternatives*” [2]
Obsolescence	“*The end point of all technology, which can progress through a lifecycle that encompasses ideas, innovation, invention, investigation, adoption, acceptance, reduced use, and obsolescence*” [21]

The following inclusion criteria were employed: studies reporting the current status of HTR activities or HTR process including the HTR agencies, candidate technology identification and priority setting, HTR methodologies, stakeholder involvement, and political support for implementation. Only studies published in English or Spanish were included.

### Study selection

Two authors independently screened the titles and abstracts according to pre-defined inclusion and exclusion criteria. The full-texts of the potentially relevant studies were retrieved to assess eligibility for inclusion, and any disagreements were resolved through a discussion with the third author.

### Data extraction and analysis

Two trained reviewers from the research team extracted the data from the articles. Subsequently, the other authors verified the extracted data. A structured data extraction form was developed to ensure uniformity and the extraction of all relevant information. This included the year of introduction of HTR, presence of legislation, agency in charge of reassessment, financial support, method of identifying reassessment candidate technology, reassessment priority setting, reassessment methodology, recommendations and decisions on reassessment results, disinvestment decision-making bodies and committee composition, implementation strategies, and stakeholder engagement. Information about HTR in four countries was summarised using descriptive methods. The results of the literature review were supplemented by data collected through interviews with informants involved in the HTR processes. Additionally, informants from the United Kingdom and Spain were visited through formal contacts in the NICE (National Institute for Health and Care Excellence) and Osteba (Basque Office for Health Technology Assessment) organisations. Informants were asked about the overall HTR policy, executive difficulties, and examples of HTR. Therefore, we attempted to clarify any missing information from the literature review. We performed a narrative synthesis rather than a quantitative one using meta-analysis to summarise and investigate the process, implementation, policies and implications for practice of HTR.

## Results

### Studies included

**Fig. 1:**
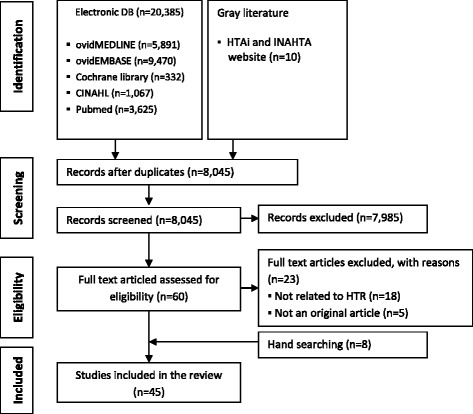
Study selection flow diagram

The electronic database and grey literature search, including the HTAi and INAHTA websites, yielded 20,395 records, of which the full text of 8045 articles were read after excluding duplicates. Sixty full text articles were retrieved and scrutinised, independently by two reviewers, as potential studies after screening the titles and abstracts. Among them, 23 articles were excluded due to the following reasons: not related to HTR programs (n = 18), and not an original article (such as letter and comments) (n = 5). Manual screening of the reference lists of included studies yielded eight additional articles. Thus, a total of 45 studies met the inclusion criteria (Fig. 1).

### HTR governance and procedures

#### Governance and organisation

The governance and performance of HTR can be affected by whether an HTR system has legal support to operate within the system. In the United Kingdom, in addition to the NICE, MaCSWise (Making Choices, Spending Wisely) was established in April 2011 as a short-term return-on-investment promotion group to focus on reassessment and reinvestment within the Scottish Health Technologies Group and National Health System [5]. In Canada, there have been initiatives aimed at prioritising, developing assessment methods for, and implementing approaches [6]. In particular, HTR has been actively implemented in Ontario, Alberta, Vancouver Island, and Calgary since the 1990s [2]. Australia has been moving forward to establish disinvestment at the local level by developing the Australasian Registry of Obsolete Health Technologies Evaluated for Disinvestment [7–9]. Spain has regulatory support on a national level, called the Royal Decree 1030 [10], while Osteba in the Basque area and the Galician Agency for Health Technology Assessment (Avalia-t) in the Galicia area play leading roles in HTR at the regional level [11, 12] (Table 2).

**Table 2:** Organisation/governance and methods of health technology reassessment in the main countries reviewed

	United Kingdom	Canada	Australia	Spain
Legal support	No	No	No	Yes
Level of performance	National	Regional	National and regional	Regional
Agency responsible	NICE	CADTH	MSAC PBAC HealthPACT Victoria, West Australia, and Queensland	Osteba Avalia-t
Methods	Health technology assessment methodology	Health technology assessment methodology, program budgeting and marginal analysis	Health technology assessment methodology	Health technology assessment methodology
Outputs	Recommendation reminder, Commissioners’ guides “Do not do” database	Recommendation	Recommendation	Report

*CADTH* Canadian Agency for Drugs and Technologies in Health, *MSAC* Medical Services Advisory Committee, *PBAC* Pharmaceutical Benefits Advisory Committee, *HealthPACT* Health Policy Advisory Committee for Technology, *Osteba* Basque Office for Health Technology Assessment, *Avalia-t* Galician Health Technology Assessment Agency, *NICE* National Institute for Health and Care Excellence

Existing HTA agencies are responsible for HTR implementation, such as the Danish Centre for Evaluation and Health Technology Assessment in Denmark [13], the Swedish Council on Health Technology Assessment in Sweden [14], and the Canadian Agency for Drugs and Technologies in Health in Canada [6]. In the United States of America, a 2008 report from the US Congressional Budget Office emphasised the importance of comparative effectiveness research as a basis for ensuring the use of emerging and expensive health technologies with superior clinical benefits compared to alternative and inexpensive health services [15]. As a result, the Patient-Centered Outcomes Research Institute has been established as an integrated public–private agency to oversee the program, although it excludes consideration of cost-effectiveness implications. Information obtained from the agency’s evaluation is expected to change technology use by promoting better decision making among consumers, the commissioner, policymakers and clinicians [13]. Establishing HTR organisations and the presence of legal support have a direct effect on policy decision-making, such as decisions on health insurance reimbursement, and influence the implementation of decisions such as discontinuing the use of obsolete health technologies in clinical practice.

#### Reassessment methodology

The HTR methodology is not very different from that of the HTA [3]. However, to overcome stakeholder resistance, HTR requires convincing evidence of at least no risk, or of a benefit, in removing the technology. Unlike evidence for new technology, providing clear evidence for the benefit or harm of established technology is more difficult. Therefore, assessments of technologies in regular use frequently depend on the analysis of routine health system data [16]. The United Kingdom’s NICE developed three strategies to support the HTR process, namely technology appraisal, recommendation reminders, and commissioning guidelines. In order to establish a basis for any decision to reduce or eliminate funding for specific technologies, a rigorous approach to evidence is required, with reliable data from high quality studies [7].

#### Identifying candidate technologies for reassessment

Most countries have similar criteria for identifying HTR candidates, which are as follows: (1) new evidence, that is, when new evidence of safety, effectiveness and cost-effectiveness is accumulated in subsequent trials, meta-analysis, post-market surveillance, audits, and registry data, geographic and providers’ variations in care are observed, temporal variations in volume are observed (e.g. 2, 3, or 5 years), there is evidence of public interest or disputes (which involves the voicing of negative (or ineffective) responses to treatment by patients or consumer advocacy and community groups), and there is conflict with guidelines or discrepancies between practices and clinical practice guidelines, the Cochrane review recommendations, etc. [17]. In the United Kingdom, the NICE health technology appraisal criteria are applied to disinvestment in a way similar to investment [18]. However, NICE might consider additional criteria. It might be relevant to establish a starting point when there is high geographical variation in healthcare service utilisation [19]. To identify treatments with little or no evidence of benefit, NICE began to use the Cochrane Library [18] and clinical practice guidelines to identify candidates for disinvestment [20] (Table 3).

**Table 3:** Criteria for the identification of health technology reassessment in the main countries reviewed

	United Kingdom	Canada	Australia	Spain
Identification	Selection criteria for medical technologies evaluation in NICE - Claimed additional benefit to patients - Claimed healthcare system benefit - Patient population - Disease impact - Cost considerations - Sustainability	- New evidence (safety, effectiveness) - Geographic variations in care - Provider variations in care (clinical heterogeneity) - Temporal variations in volume (a trend in item volume between time points) - Technology development - Public interest or controversy - Consultation - Nomination - Assess new intervention, displace old - Leakage - Legacy items - Conflict with guidelines - Precedent	- New evidence (safety, effectiveness) - Geographic variations in care - Provider variations in care (clinical heterogeneity) - Temporal variations in volume (a trend in item volume between time points) - Technology development - Public interest or controversy - Consultation - Nomination - Assess new intervention, displace old - Leakage - Legacy items - Conflict with guidelines	Guideline for Not Funding existing health Technologies in healthcare systems - Technology to be used in the centre or place - Technology status known to the applicant - Alternative treatment option available - No absence of care with disinvestment
				Detection criteria - Is currently used (health services portfolio) - Alternative technologies are available

In the United States, the Institute of Medicine developed a topic list of priorities for technology reassessment and for the development of candidate technologies for reassessment. Similar to other countries, its identification of candidate technologies for reassessment involves searches of unnecessary, ineffective or harmful interventions, and the systematic investigation of variations in clinical practices. The Institute of Medicine’s candidates for potential disinvestment are existing health technologies that show a decreased frequency of use because of problems regarding their safety and effectiveness [13].

#### Prioritisation of candidate technologies

Several countries shared common prioritisation criteria for disinvestment, including the following: the cost of the technology has a major impact on the total budget; the benefit of technology is small [7]; the cost per procedure and by volume is high; low degree of disease or burden, especially in vulnerable populations [17]; experience-based regional requests and decisions; new evidence on safety, effectiveness and cost-effectiveness; and time-based criteria (e.g. approval of new health technologies and reassessment 5 years after introduction) [21].

In Canada, the “Oversight Committee” model was proposed to consider the priority-setting process and strategies to identify potentially obsolescent technologies [21]. In Spain, the PriTec tool developed by Avalia-t is used for HTR candidate priority setting, considering population/end-user factors, a risk-benefit analysis and costs, as well as organisational factors and other implications [22] (Table 4).

**Table 4:** Criteria for prioritisation of health technology reassessment in the main countries reviewed

	United Kingdom	Canada	Australia	Spain
Prioritisation	- Budget impact - Existing alternatives - Improved patient safety - Not for vulnerable populations - Small benefit - Close risk/benefit ratio	- Cost of service - Potential impact - Cost-effective alternative - Disease burden - Sufficient evidence available - Scope for time-limited funding with “pay for evidence” or “only in research” provisions - Futility	- Cost of service - Potential impact - Cost-effective alternative - Disease burden - Sufficient evidence available - Scope for time-limited funding with “pay for evidence” or “only in research” provisions - Futility	Population/users - Disease frequency - Burden of disease - Frequency of technology use - Patients preferences Risk/benefit - Efficacy/effectiveness/validity - Adverse effects - Risks Costs, organisation, other implications - Efficiency - Maintenance costs - Other implications

#### Stakeholder involvement and decision-making processes for disinvestment

The decision-making process for reassessment should be transparent and supported by robust evidence, and implementation should include appropriate knowledge transfer to all stakeholders [16]. In order to expedite the acceptance of individual disinvestment decisions, the decision-making process should be conducted in cooperation with patients, who are given sufficient information regarding the reasons for the decision. The health professionals who use the technology should also be informed of the reasons underpinning the decision and be involved in the process of identifying and evaluating whether a technology is suitable for disinvestment [23].

In the United Kingdom, the body for decision making on disinvestment is the Technology Appraisal Committee. Committee members are drawn from the National Health Service, patient and caregiver organisations, academia, and pharmaceutical and medical device industries. Commentator organisations include the manufacturers of comparator technologies, National Health Service Quality Improvement Scotland, the relevant National Collaborating Centre (a group commissioned by NICE to develop clinical guidelines in areas such as cancer and mental health), and research groups working in the area [24]. In Vancouver, Canada, a standard approach to Programme Budgeting and Marginal Analysis (PBMA) was taken with a priority-setting working committee that comprised of all directors and clinical leaders from Vancouver communities, as well as a broader advisory panel that includes a mix of personnel and senior executive members from Vancouver communities. The characteristics of the decision-making process include the use of a standard business case template and proposal rating tool, an assessment standard with clearly defined weighted values related to the health authority’s strategic priorities, and a public communication plan. Through several deliberations, the research teams provide the modified and refined standard to the operating committee [25]. Canada’s policy decision-making process included removal from funding schedules, partial reimbursement, and risk-sharing with the health service provider reimbursing the agreed cost to the insurer. If the outcomes are below expectations, reimbursement is given only for guideline adherence, and there are sunset clauses in financial support regulations, which stipulate that reimbursement is provided on the condition that proper periods are set and evidence generated [6]. In Australia, deliberative democratic methods were adopted to develop evidence-informed stakeholder engagements that include clinicians, consumers and representative community members in the process. The community consultations were held over 2 days to allow for information sharing, deliberation and an understanding of the HTR reports [26].

Spain’s disinvestment decision-making process follows the Guideline for Not Funding existing health Technologies in health care systems (GuNFT), announced in May 2010. The decision-making results include eight possibilities: in favour of the proposal; against the proposal; against the proposal, but modifiable in the future; against the proposal because of a lack of evidence, but modifiable in the future; proposal recommended; proposal not recommended; proposal not recommended, but can be considered when the capacity of the centre has been revised; and proposal not recommended, but modifiable when new evidence is available [12]. Information asymmetry between clinically engaged experts and policy or decision makers may preclude the truly collaborative, informed and technical discussions required to generate genuine change. Overcoming these challenges will require innovative approaches to co-producing evidence syntheses, broad-based stakeholder engagements, and a sustained commitment from clinicians and policymakers alike [27].

While PBMA is being used to assess the distribution of resources for health services and technologies within a fixed budget plan, HTA review is mainly focused on specific technologies in the fee-for-service healthcare payment system [28].

#### HTR implementation

There appear to be several main barriers to HTR implementation [16]. First, patients and clinicians tend to think that the decision to remove an existing health technology poses a greater disadvantage than the decision to not accept a new health technology with a similar value. Second, professional or system inertia is a particular challenge to overcome. Introducing change to existing technology is difficult since trained clinicians consider technology integral to their professional practice and identity. The clinical training and practice paradigms can be difficult to change, and organisations could have invested a considerable amount of money in the existing technology and capital infrastructure. Third, to overcome stakeholder resistance, convincing evidence of no harm from withdrawal and no benefit from technology use is required. Sometimes stronger evidence is needed for use reduction or withdrawal decisions than for other aspects of technology use.

In the United Kingdom, a recommendation reminder is published monthly, summarising new recommendations for the use of existing health technologies. The “Do Not Do” list, recommendation reminders and Commissioners’ guides issued by the NICE list all of the health technologies that the organisation suggests avoiding or not using [20]. The Canadian Institute of Health Economics utilises the Ambassador Program for Knowledge Transfer as a representative program and is trying to disseminate reassessment results through Consensus Development Conferences [29]. In Spain, the GuNFT was developed as a free software package to facilitate quick and easy communication between stakeholders who are participating in the process of disinvestment. This software sends emails containing applications for technologies targeted for disinvestment to assessors and decision makers. The reassessment process and results report are provided after the evaluation process has been completed [12].

### Suggestions to facilitate HTR in experienced countries

Decisions on disinvestment and resource allocation required deliberative process for ensuring transparency and objectivity. It needs to articulate definition of disinvestment for various stakeholders because disinvestment may be understood differently depending on their conflict of interests. To boost decision-making on disinvestment, training for resource reallocation methods was provided to those who participate in panel advisory groups. Additionally, contextual and colloquial evidence as well as clinical evidence need to be obtained and collated to facilitate in-depth discussion in decision-making regarding disinvestment. Moreover, political support, including political motivation, transparency and governance, might underpin the introduction and establishment of HTR.

Specific examples on disinvestment from experienced countries were as follows. An investigation had been conducted on those who were participating in local-level commissioning meetings in terms of how they understand disinvestment and how they implement disinvestment using ethnographic methods. Most informants, including commissioners, hospital managers and lay members, had described disinvestment as funding reduction or withdrawal from the existing healthcare services, whereas some participants reported disinvestment as the main motive of cost-saving. These comments indicated that it was necessary to provide a clear definition of disinvestment. Further, they pointed out that, while the lack of information, guidance and time capacity were the practical barriers for disinvestment, difficulties for collaboration between commissioners and hospital managers/clinicians due to distrust were ideological barriers. Therefore, it would be necessary to encourage hospital managers, clinicians and lay members, whose disinvestment was to be potentially affected, to participate in the decision making regarding disinvestment, including defining clearly what disinvestment would be, and to develop the strategies for an explicit disinvestment agenda in consideration of the barriers investigated [30].

A study on stakeholders who had used PBMA for resource reallocation of Vancouver Island Health Authority in Canada reported that two tasks needed to be achieved before introducing a new priority-setting process in healthcare organisations. The first task concerned the procedures addressing the needs of strategies to educate and train a panel advisory group since the senior decision-makers presented a learning curve as they became familiarised to the PBMA approach in the early stage of PBMA implementation. The second task was to establish the basic principles of decision making on redistribution, evidence-based decision making methods and transparent decision making processes to explain funding decisions [31].

In Australia, a case study on the use of assisted reproductive technologies (ART) for older women asked physicians who participated in the process of deliberative stakeholder engagements about the evidence needed to assess the eligibility of ART as a disinvestment candidate. The physicians had criticised currency, proximity, selectivity and bias for statistics drawn from the Australian and New Zealand Assisted Reproduction Database, a registry of the outcomes of all initiated cycles of ART. Thus, the participatory policymaking process of stakeholders claimed the need for various empirical (what are the ‘real’ outcomes of ART?), contextual (contextual factors impacting on ART outcomes), and anecdotal evidences (data related to patient behaviours) rather than traditional research evidence to enable sufficient discussion at the time of decision making for disinvestment [28, 32].

In a study investigating the barriers related to the establishment of an agency to perform research that generates evidence on the funding or selective disinvestment of the Spanish National Health System, a panel of experts rated political domains as the main barriers among the lack of political motivation, tension between a decentralised health system and evaluation activities, technical difficulties of the HTR processes or decision-making, and social and professional reluctance for the withdrawal of healthcare benefits [33]. Therefore, it was found that there was room for improvement to incorporate explicit mechanisms for decision-making on disinvestment of health technologies in the Spanish national health reimbursement system [34].

## Discussion

From our review of the literature from various countries, we suggested actions that may be taken to improve the Korean HTR system. Firstly, a formulary legal structure for HTR was needed in order to increase the impact of HTR system introduction. The HTA program was introduced in Korea in 2007, focusing on the new HTA system of Decree 53 in the Medical Service Act. Therefore, the existing Medical Service Act could be revised by adding the tasks related to HTR.

The second is related to the criteria for the identification and prioritisation of HTR; in most countries, the identification of candidates for HTR was performed by direct searching of biomedical studies, HTA reports, clinical practice guidelines and new health technology databases, and by contacting clinical experts. The common criteria were efficiency, effectiveness, cost-effectiveness, safety, regional requirement or patient preference, existence of available substitute, and the cost and burden of the disease. In Korea, seven “identification” criteria, seven “prioritisation” criteria and weighted values, and four “reassessment” criteria to enable the practical execution of reassessment were selected using the RAND Method. The “identification” criteria included safety, controversial efficacy, proposal from Department of Health, Korean Medical Association and patient related associations, new evidence on effectiveness, significant changes in the utilisation frequency of technologies, new findings of cost-effectiveness of technologies, and variation of regional or providers’ provision. The “prioritisation” criteria included safety, efficacy, volume on evidence, burden of diseases, potential impact of reassessment, utilisation of technologies and cost-effectiveness. Finally, the reassessment criteria for HTR included safety, efficacy or effectiveness, efficiency or cost-effectiveness, and infrastructure of the healthcare system.

Thirdly, HTR methods have been largely based on the HTA methodology. However, HTR has additional requirements such as no harm due to withdrawal of health technologies, or strong evidence that the use of the technology has no benefit. Furthermore, HTR needs to include the systematic assessment of social value, patient preference and ethics. Therefore, in order to generate convincing evidence for the disinvestment of existing technologies, a new national registry for HTR should be created to investigate the influence of health technologies on health outcomes, as well as to analyse existing national health insurance claims data from the Health Insurance Review & Assessment Service and National Health Insurance in Korea.

Finally, the Korean HTR system might classify the top-down programs initiated by governmental authorities. The binding clinical practice guideline or reimbursement in coverage may facilitate the uptake of implementation of HTR, whereas it can raise resistance of physicians and consumers related to technologies rated as withdrawal decision [35]. Therefore, it needs to make a decision underpinned by the varied involvement and active communication of physicians and various stakeholders, including patients, insurers and health authorities.

**Fig. 2:**
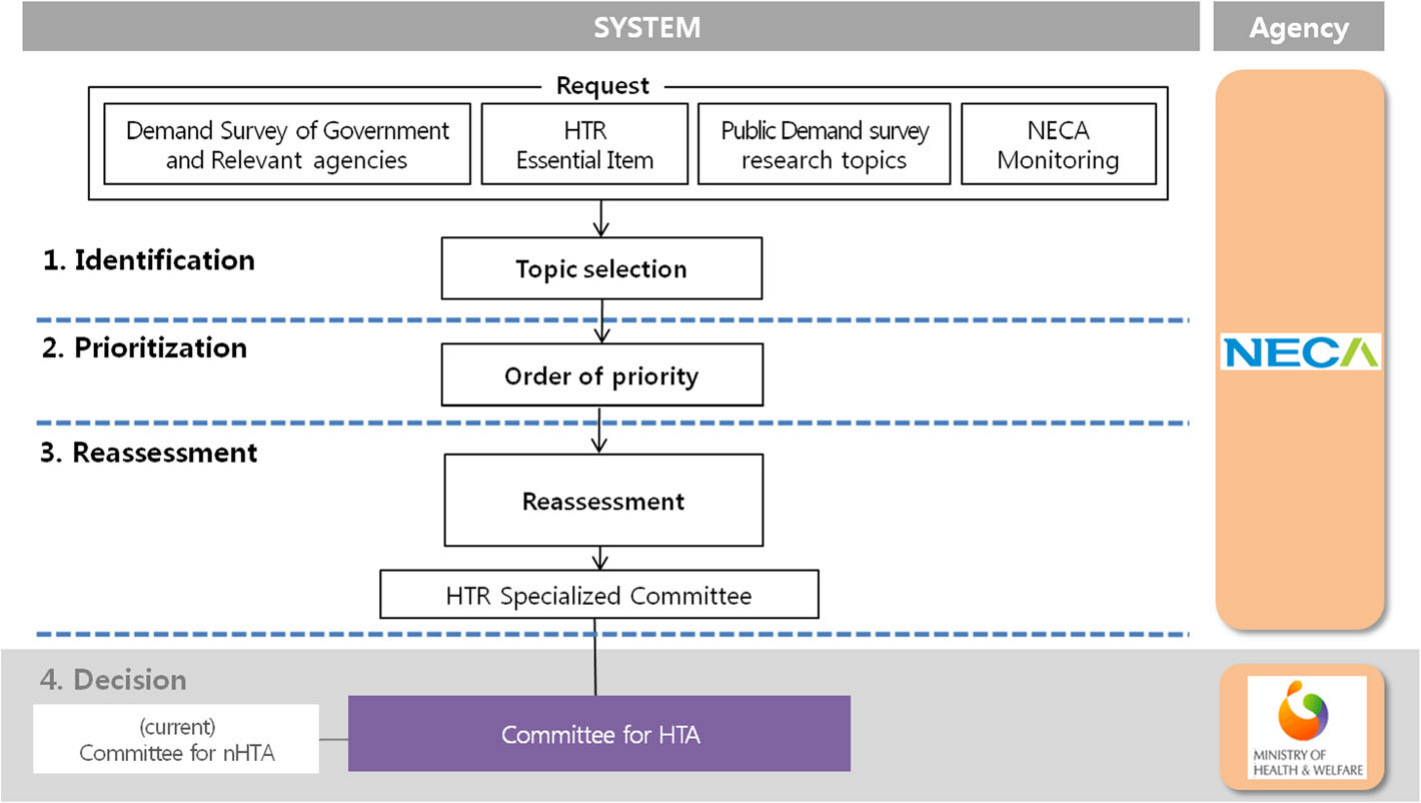
Health technology reassessment process in Korea

Meanwhile, in South Korea, two HTR pilot projects began on April 1, 2014, which were expected to continue for 2 years. The aims of the pilot projects were to assess feasibility of the introduction and implementation of HTR program, to refine the objectives of the HTR for reimbursement coverage or (partial/complete) disinvestment, and to set prioritisation criteria according to objectives of HTR program. Considering the findings of this systematic review and the domestic healthcare environment, we developed a four-stage Korean HTR model, including “identification”, “prioritisation”, “reassessment” and “decision.” First, in terms of identification, regarding health technology information collected through a demand survey conducted by a Government ministry, related agency and specialised society, or internal monitoring by the NECA, the identification criteria already determined are applied to identify the medical technology eligible for reassessment. Second, in the prioritisation stage, the reassessment of the selected health technologies is prioritised based on predetermined prioritisation criteria and weighted values. Third, for the finally selected health technology, methodologies, such as systematic review and economic feasibility analysis, are used based on the reassessment criteria to execute the HTR. Here, the specialised reassessment committee (provisional name) provides the mediation plan based on the details discussed. Finally, in the decision stage, the Health Technology Assessment Committee (currently, Committee for nHTA) finally decides and notifies the reassessment result on the relevant health technology based on the mediation plan advice (Fig. 2).

This reassessment model was implemented on intestine capsule endoscopy and intra-disc steroid injections. The research group plan to collate the data and publish a progress report and evaluation of the HTR projects. This will demonstrate Korea’s experience with HTR at the national level.

This review has some limitations. First, despite the fact that our review included studies from seven different countries, we excluded articles written in languages other than English or Spanish. Second, the included studies were collected by searching electronic databases and HTAi member agencies rather than by contacting experts and researchers in each country, with the exception of Spain and England. Nevertheless, this was a comprehensive review of the literature on HTR, reporting implications and practical information from other countries to help introduce HTR in Korea.

## Conclusion

The current study reviewed HTR system, focusing on the United Kingdom, Australia, Canada and Spain. There were similarities in the HTR processes, including utilisation of existing HTA agencies, identification and priority setting of candidate technologies for reassessment, reassessment methods, and the composition of advisory committees including various stakeholders.

Disinvestment through HTR was one of the strategies to increase the efficiency and quality of care to help patients receive optimal treatment. Based on the lessons from foreign countries’ experiences, South Korea should make efforts to develop and establish an HTR system to optimise the National Healthcare Insurance system.

## Abbreviations

Avalia-t: Galician Agency for Health Technology Assessment
GuNFT: Guideline for Not Funding existing health Technologies in health care systems
HTA: health technology assessment
HTAi: Health Technology Assessment International
HTR: health technology reassessment
INAHTA: International Network of Agencies for Health Technology Assessment
MaCSWise: Making Choices, Spending Wisely
NECA: National Evidence-based healthcare Collaborating Agency
NICE: National Institute for Health and Care Excellence
Osteba: Basque Office for Health Technology Assessment
PBMA: programme budgeting and marginal analysis
